# CaMKII Activity in the Inflammatory Response of Cardiac Diseases

**DOI:** 10.3390/ijms20184374

**Published:** 2019-09-06

**Authors:** Maria Rosaria Rusciano, Elena Sommariva, Victorine Douin-Echinard, Michele Ciccarelli, Paolo Poggio, Angela Serena Maione

**Affiliations:** 1 Department of Medicine, Surgery and Odontology, University of Salerno, 84081 Baronissi, Italy (M.R.R.) (M.C.); 2 Casa di Cura Montevergine, 83013 Mercogliano, Italy; 3 Vascular Biology and Regenerative Medicine Unit, Centro Cardiologico Monzino IRCCS, 20138 Milan, Italy; 4 Institute of Cardiovascular and Metabolic Diseases, Inserm UMR 1048, 31432 Toulouse, France; 5 Paul Sabatier University, 31432 Toulouse, France; 6 Unit for the Study of Aortic, Valvular and Coronary Pathologies, Centro Cardiologico Monzino IRCCS, 20138 Milan, Italy

**Keywords:** CaMKII, inflammation, Ca^2+^, ROS, NF-κB, cardiac diseases

## Abstract

Inflammation is a physiological process by which the body responds to external insults and stress conditions, and it is characterized by the production of pro-inflammatory mediators such as cytokines. The acute inflammatory response is solved by removing the threat. Conversely, a chronic inflammatory state is established due to a prolonged inflammatory response and may lead to tissue damage. Based on the evidence of a reciprocal regulation between inflammation process and calcium unbalance, here we described the involvement of a calcium sensor in cardiac diseases with inflammatory drift. Indeed, the Ca^2+^/calmodulin-dependent protein kinase II (CaMKII) is activated in several diseases with an inflammatory component, such as myocardial infarction, ischemia/reperfusion injury, pressure overload/hypertrophy, and arrhythmic syndromes, in which it actively regulates pro-inflammatory signaling, among which includes nuclear factor kappa-B (NF-κB), thus contributing to pathological cardiac remodeling. Thus, CaMKII may represent a key target to modulate the severity of the inflammatory-driven degeneration.

## 1. The Immune System and the Inflammatory Process in the Heart

Inflammation is a natural and necessary immune reaction that occurs when organisms experience infections, stress, or tissue damage to fight the insulting agent. Although essential for body protection against pathogens, excessive inflammation can provoke by-stander injury and cause organ dysfunction [1]. Inflammation is a complex process ensuring leukocyte infiltration at the site of tissue injury, and it is finely tuned by a large panel of molecules, tissue resident immune cells, and stromal cells [2]. 

Typically, the inflammatory reaction is composed of four constituents: inducers of inflammation; sensors on the cell surface that detect them; mediators, produced when prompted by the sensors; and the target tissues that respond specifically to the inflammatory mediators. Different forms exist for each constituent, and their combinations compose distinct inflammatory pathways. The type of pathway induced depends on the nature of the trigger [1]. 

Pathogens are recognized by several major classes of pattern recognition receptors (PRRs), expressed both in immune and non-immune sentinel cells, which are activated by pathogen-associated molecular patterns (PAMPs) [1]. 

Sterile inflammation takes place in the absence of pathogens. In this case the trigger is constituted by intracellular particles released by necrotic or apoptotic cells. In this case PPRs are activated by endogenous agents (danger-associated molecular patterns; DAMPs) to elicit an inflammatory response [3].

PRRs include the Toll-like receptors (TLRs), C-type lectin receptors (CLRs), retinoic acid-inducible gene (RIG)-I-like receptors (RLRs), and NOD-like receptors (NLRs) [4]. 

Receptor activation triggers relevant intracellular signaling pathways, among which are the mitogen-activated protein kinase (MAPK), nuclear factor kappa-B (NF-κB), Janus kinase, activator protein-1 (AP-1), interferon regulatory factor 3 (IRF3), and activation of transcription [5,6]. 

Gene transcription activation drives, in turn, the production and secretion of pro-inflammatory cytokine, such as interleukin (IL)-1, tumor necrosis factor (TNF)-α, IL-6, colony stimulating factor (CSF), interferons, transforming growth factor β (TGF-β), and chemokines, which contribute to the inflammatory response [7].

The acute phase of the response is characterized by a massive influx of granulocytes, then monocytes, which both play a predominant role in the clearance of the pathogen and removal of tissue debris. 

The resolution of inflammation is tightly regulated [8]. The severity of disease pathogenesis may be related to effective resolution or chronicization of the inflammatory process [9,10]. 

In particular, a chronic inflammatory process plays a crucial role in the progression of heart diseases and exerts a deleterious role on cardiac function. Heart specific cytokines, neurohormones and pro-inflammatory molecules, which can be referred to as cardiokines, actively drive the progression of cardiac dysfunction in heart failure [11,12]. The cells composing the heart, such as cardiomyocytes, fibroblasts, vascular cells, and progenitor cells, are able to secrete several cardiokines following different environmental stimuli, realizing a specialized network that is critical for heart homeostasis. These proteins, including cytokines, such as TNF-α and TGF-β or different interleukins, are able to control the balance between normal cardiac function and pathological myocardial remodeling based on their ability to influence cardiomyocyte apoptosis, fibroblast activation, and vascular cell proliferation. 

Notably, low concentrations of TNF-α produces a cardioprotective effect, while increased levels of TNF-α have been associated to heart failure and diastolic dysfunction, and is positively correlated to the severity of the diseases. Transgenic mice with a cardiac-specific TNF-α overexpression display heart failure, cardiac dilatation, fibrosis, altered contractile function, Ca^2+^ handling defects, and premature death [13]. Furthermore, the progression of TNF-α-induced cardiac remodeling is associated to the activation of cardiomyocyte apoptosis and proteasome dysfunction [14,15]. The TNF-α increase occurring during ischemia/reperfusion injury (I/R) is related to Ca^2+^ overload and the resultant cardiac dysfunction [16,17]. The pharmacological modulation of TNF-α production is able to improve cardiac function and reduce the intracellular Ca^2+^ overload and oxidative stress that arises following I/R stress [17,18,19].

In addition, TGF-β, another cardiokine that also has a physiological cardioprotective effect, if deregulated, actively participates in the pathological cardiac remodeling mediating the tissue fibrosis that follows the tissue-injury-derived inflammation acting on fibroblast activation, differentiation, and extracellular matrix protein secretion [20,21]. TGF-β1 over-expression has been associated with myocardial hypertrophy, hypertensive cardiac remodeling, several cardiomyopathies, and genetic aortic syndromes [22,23]. Transgenic mice with the TGF-β type 2 receptor conditional knockdown in cardiomyocytes display, following a sustained pressure condition, reduced interstitial fibrosis and improved heart function, and they do not exhibit cardiac dysfunction and chamber dilation [24]. The TGF-β1-dependent cardiac fibrosis also correlates to the regulation of intracellular Ca^2+^ concentrations by the type 2 ryanodine receptor (RyR2). TGF-β1 and collagen levels are up-regulated in cardiomyocytes subjected to mechanical stress, but this event is reverted in RyR2 knockdown cardiomyocytes [25]. Pharmacological inactivation of non-canonical TGF-β signaling by arjunolic acid treatment leads to the up-regulation of peroxisome proliferator activated receptor alpha and results in the down-regulation of collagen gene expression in the hypertrophy-model of cardiac fibroblasts [26].

Recently, an emerging role for the IL-33/ST2 pathway in the inflammation that occurs during the cardiac stress condition has been described [26,27]. ST2, which belongs to the Toll-like receptor family, exerts an immunomodulatory effect based on its ability to regulate cytokine production [28,29]. Furthermore, the soluble ST2 form represents a predictive biomarker in patients with chronic heart failure and a severe prognosis [29]. The IL-33/ST2 interaction results in an anti-hypertrophic effect by blocking NF-κB activation. Mice lacking ST2 have worsened hypertrophy, cardiac dilation, ventricular fractional shortening, increase fibrosis, and reduced survival in a pressure overload condition [30].

## 2. Calcium/Calmodulin-Dependent (CaMK) II in the Heart

Calcium/calmodulin-dependent kinases are a family of serine/threonine kinases that respond to the intracellular calcium Ca^2+^ changes [Ca^2+^]_i_ and consist of three members: CaMKI, CaMKII, and CaMKIV [31]. Ca^2+^ transduces its functions by forming a complex with calmodulin (CaM), which acts as a ubiquitous Ca^2+^ receptor [31]. 

CaMKII is a multimeric enzyme consisting of 12 monomers [32]. Each monomer shares the same structure that consists of an N-terminal catalytic domain, a C-terminal association domain, and the central auto-regulatory domain where the Ca^2+^/CaM binding site is located [33]. CaMKII is the most suitable decoder of total [Ca^2+^]_i_ although it is also engaged by intracellular Ca^2+^ oscillations and transients [34,35].

Under resting conditions, the CaMKII regulatory domain interacts and sterically blocks the catalytic domain, leading to its auto-inhibitory state. The activation process requires the binding of the Ca^2+^/CaM complex, which displaces the intrasterical auto-inhibition and exposes the kinase substrate and ATP binding sites of the catalytic domain [36]. At this point, the activated monomer is able to sequentially phosphorylate at Thr286/287 (depending on CaMKII isoforms), the regulatory domains of adjacent CaMKII monomers. The auto-phosphorylation confers to CaMKII an autonomous kinase activity even after the dissociation of the Ca^2+^/CaM complex, thus preventing the re-association of the catalytic domain with the auto-inhibitory domain [33,37]. Furthermore, the activation also induces the “CaM trapping” that leads to an increased affinity to CaM binding and to a time-sustained CaMKII activity upon low [Ca^2+^]_i_ conditions [38].

An alternative route of CaMKII activation has been described involving the reactive oxygen species (ROS) produced by various sources including nicotinamide adenine dinucleotide phosphate (NADPH) oxidase and mitochondria. Specifically, ROS oxidizes CaMKII, at methionine 281/282, which remains active even in the absence of the Ca^2+^/CaM complex [39]. Essentially, the oxidation of the methionine residues of CaMKII works as a sensor of ROS increments and correlates with a sustained kinase activity [39,40].

Another possible trigger of CaMKII autonomous activation is hyperglycemia. The extracellular glucose elevation leads to *O*-linked *N*-acetyl-glucosamine (*O*-GlcNAc) modification at CaMKII S279 [41]. Furthermore, CaMKII autonomous activity can be induced through a nitric oxide (NO)-dependent pathway by *S*-nitrosylation of Cys290 [42]. Notably, both *O*-GlcNAc and *S*-nitrosylation modifications require the initial Ca^2+^/CaM-dependent activation and result in persistent autonomous CaMKII activation [41,42].

The CaMKII inactivation involves either phosphatase-dependent or -independent mechanisms. The dephosphorylation of Thr286 occurs through 70% of the protein phosphatase 2A (PP2A) activity; PP1 and PP2C act for the remaining activity [43]. An alternative CaMKII inactivation mechanism, which is typical of post-synaptic plasticity regulation [13], consists of the auto-phosphorylation of Thr305/306 that prevents the CaM rebinding to the regulatory domain (CaM-capping) [33] by modifying the Ca^2+^/CaM binding site [44,45].

The CaMKII tissue distribution is variable, and the four CaMKII isoforms (α, β, γ, δ) encoded by separate genes show tissue-preferential expression [46]. CaMKIIα and β are the neuronal isoforms [47] while the CaMKIIδ and γ isoforms are predominantly expressed in cardiac tissue [48,49]. 

CaMKIIδ is critical during the pathogenesis of cardiac hypertrophy after catecholaminergic stimulation [50]. It modulates transcription by mediating histone deacetylase (HDAC)4 phosphorylation during pressure overload [51]. CaMKIIδ affects Ca^2+^ handling by phosphorylation of RyR2 and phospholamban (PLN), thus inducing changes in sarcoplasmic reticulum (SR) Ca^2+^ content and resulting in diastolic Ca^2+^ leak [51], leading to diastolic dysfunction and arrhythmogenesis [52].

Several pieces of evidence correlate CaMKII activity to physiological functions such as cell proliferation and cell cycle progression. In particular, the inhibition of CaMKII reduces vascular smooth muscle [53] and endothelial cell proliferation [54] as well as S-phase progression of the cell cycle [55]. On the other hand, the over-expression of CaMKIIγ negatively regulates vascular smooth muscle proliferation [56]. Interestingly, CaMKII specific inhibitors increase proliferation of cardiomyocytes derived from induced pluripotent stem cells [57].

## 3. CaMKII and Inflammation in Cardiac Diseases

Ca^2+^ has been associated with different events of the inflammatory response [58,59,60,61] as well as with the regulation of proliferation, anergy and cell death of T cells [62]. Based on its ability to act as an intracellular Ca^2+^ sensor, CaMKII is recognized as a key regulator of the immune and inflammatory responses [63,64,65] at different levels.

CaMKII regulates the physiology of T cells. The Ca^2+^-independent form of CaMKIIγ enhances T cell memory formation and modulates cell death [66]. In T cells, treatment with CaMKII inhibitor KN93 modulates the NF-κB activation pathway by abolishing the phorbol-ester-induced phosphorylation of inhibitory κB (IκB) proteins [67]. Moreover, CaMKII modulates IL-10, IL-2, and IL-4 production by T lymphocytes. Specifically, the overexpression of the constitutively active CaMKII form leads to increased IL-10 protein and mRNA accumulation based on its ability to directly modulate IL-10 promoter activity [68]. In addition, it is involved in the Ca^2+^-dependent IL-2 transcriptional arrest, causing anergy [63,64], and regulates IL-4 by direct action on its promoter [63]. 

Studies performed on macrophages highlighted that CaMKII boosts pro-inflammatory cytokines and type I interferon production upon TLR stimulation [69] and participates in the Wnt5A signaling-mediated inflammatory response [70]. 

Furthermore, CaMKII regulates dendritic cell physiology, acting at different levels on the expression and localization of MHC Class II proteins [71], cell maturation, and the antigen presentation ability following phagocytosis-induced stimulation [72].

**Figure 1:**
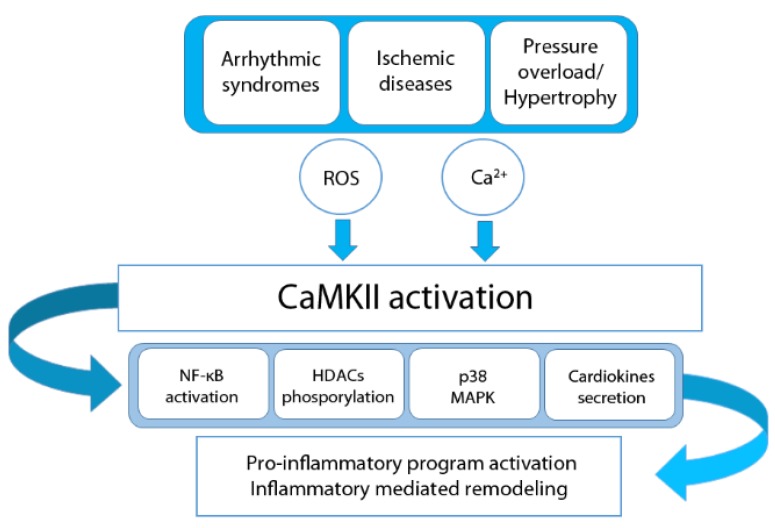
Schematic description of Calcium/calmodulin-dependent (CaMK) II involvment in the inflammatory response in cardiac diseases.

In addition to the response to pathogens, the inflammation process in cardiac disease is mostly an adaptive response to myocardial injury [73,74]. In particular, sustained CaMKII activation is demonstrated to be involved in several cardiovascular diseases. Notably, the inhibition of CaMKII has been suggested as a novel therapeutic target to treat cardiac arrhythmias, heart failure, and hypertrophy [75,76,77]. The following subsections will summarize the main findings and mechanisms regarding cardiac diseases in which CaMKII and inflammation mediate pathological remodeling (Figure 1).

### 3.1. Ischemic Diseases

The cytosolic Ca^2+^ overload is one of the common events that leads to heart failure and ischemic heart disease. The resulting sustained CaMKII activation promotes L-type Ca^2+^ channel opening probability by phosphorylation of the α-subunit of L-type voltage-gated Ca^2+^ channel (CaV1.2). L-type Ca^2+^ channel opening regulates cellular Ca^2+^ homeostasis, which in turn controls cardiac myocyte apoptosis [78]. In vivo, CaMKII inhibition is able to protect against myocardial apoptosis induced by myocardial infarction [79] and restores SR Ca^2+^ content. The RyR2 mutated mouse model, which lacks the CaMKII phosphorylation site, is resistant to apoptosis and displays improved cardiac function after myocardial infarction [80]. In addition, the overexpression of mutant CaV1.2, which is resistant to CaMKII binding and thus precludes CaV1.2 phosphorylation, retards cardiomyocyte death [81]. Moreover, the overexpression of CaMKIIδ leads to increased cardiomyocyte apoptosis, together with elevated cytosolic Ca^2+^ and enhanced mitochondrial cytochrome C release [82].

Moreover, ischemic-induced necrotic cell death with consequent release of intracellular molecules [83] results in the activation of TLRs in cardiomyocytes, inducing pro-inflammatory transcriptional pathways [84,85,86,87]. Several pieces of evidence have established that CaMKII has a central role in regulating inflammation in myocardial infarction (MI), since it is oxidized as a consequence of increased β-adrenergic activation upon MI, which is followed by increased intracellular ROS [88,89]. The oxidized CaMKII is able to enhance pro-inflammatory transcriptional signaling by promoting NF-κB activity [67]. Gene expression profiling performed in mouse hearts of transgenic AC3-I mice, in which there is a cardiomyocyte-limited expression of a CaMKII inhibitory peptide, showed that CaMKII inhibition reduces the post-MI upregulation of pro-inflammatory genes and complement factor B [90].

The inflammatory response also occurs during cardiac reperfusion following an acute ischemic event. In addition to the pro-inflammatory signaling-activated cardiomyocyte death described for MI, I/R injury also leads to the opening of the mitochondrial permeability transition pores, resulting in the increase of cellular Ca^2+^ and ROS [91,92]. It has been demonstrated that cardiac-specific CaMKIIδ deletion protects against I/R since it decreases infarct size, attenuates apoptosis, and improves functional recovery. CaMKIIδ deletion is also able to reduce I/R-induced inflammation by preventing the reduction of IκB and upregulation of NF-κB target genes [93]. In contrast, in a similar I/R study, an effect on infarct size following I/R CaMKIIδ KO, CaMKIIγ KO, and CaMKIIγ/δ double knockout (DKO) mice has not been observed. A reduced infarct size and improved cardiac function are observed only at five weeks after I/R in CaMKIIγ/δ DKO mice. Notably, loss of CaMKII reduces the cardiomyocyte expression and secretion of the chemokines C-C motif ligand 2 and 3, leading to decreased infiltration of CD45^+^ leukocytes, thus attenuating inflammatory mediated post-infarct remodeling [94].

### 3.2. Pressure Overload/Hypertrophy

The recruitment of immune cells due to inflammatory responses and contribution to cardiac remodeling also occurs with pressure overload [95,96,97]. Angiotensin II (Ang II) infusion represents the common treatment to study the inflammatory-induced remodeling by hypertensive non-ischemic stress [98]. It has been reported that Ang II treatment induces NF-κB-dependent inflammatory gene expression and inflammasome activation, which were reduced in a cardiomyocyte-specific CaMKIIδ KO mouse model. Therefore, CaMKIIδ activation mediates inflammation-driven remodeling [99]. As an alternative mechanism, Ang II promotes ROS release, the oxidation of CaMKII, thus resulting in the activation of p38 MAPK, another major mediators of the inflammatory response [100].

An alteration of intracellular Ca^2+^ cycling has also been observed in another experimental model of pressure overload, the transverse aortic constriction (TAC), which reflects increased afterload [101,102]. In turn, afterload is responsible for CaMKII activation based on the induced L-type calcium current increase [103,104]. The TAC model results in hypertrophy with increased fibrosis, inflammation, cardiomyocyte apoptosis, and persistent CaMKII activation [102]. As described for other models, CaMKIIδ activation triggers the inflammasome through NF-κB and ROS signaling in cardiomyocytes, inducing chemokine production, which contributes to macrophage infiltration and the development of fibrosis [105]. Likewise, fibrosis and ventricular dilation and dysfunction can be reduced by both selective CaMKIIδ deletion and by blocking CaMKII activation within the first two weeks of TAC and after the onset of inflammatory cell accumulation [105], thus confirming the CaMKII involvement in the maladaptive response during pressure overload [106].

It has been reported that CaMKII is involved in the transcriptional regulation of hypertrophic genes by regulating the phosphorylation of histone deacetylases (HDACs), which in turn affect TNF-α and IL-1β expression and cardiac fibrosis [107,108]. CaMKII is able to phosphorylate and prevent the nuclear import of HDAC4, based on the presence of two conserved CaMK phosphorylation sites in the N-terminal regions of class II HDACs, thereby inducing the repression of MEF2 and the activation of the hypertrophic program [108,109]. Mice lacking the δ isoform of CaMKII display a reduced phosphorylation of HDAC4 and are protected against hypertrophy and fibrosis following TAC [48]. Analogously, hypertrophic genes such as ANF, brain natriuretic peptide, myosin heavy chain, and skeletal actin are overexpressed in the heart of transgenic mice with cardiomyocyte-specific expression of CaMKIIδB and CaMKIIδC due to the induced transactivation of MEF2 [51]. A common event occurring during hypertrophy is the increase of the systemic levels of Ang II, which in turn acts as an activator of cardiac fibroblast proliferation. The excessive cardiac fibroblast proliferation is associated with inflammatory cytokine secretion and promotes the progression of cardiac fibrosis, thus contributing to the heart failure. The inhibition of CaMKII is able to reduce the Ang-II-induced cardiac fibroblast proliferation as well as the secretion of TGF-β1 and TNF-α. Moreover, CaMKII inhibition also reverts the upregulation of MMP-1, 2, and 9 and collagen I and III following Ang II treatment, confirming its involvement in extracellular matrix regulation [110].

CaMKII also acts as downstream target of β-adrenergic receptor (βAR) signalling. The cytosolic Ca^2+^ increase, following βAR stimulation, is related to the physiologic augment of cardiac contraction. Excessive βAR activation results in pathological heart remodeling and myocardial hypertrophy. A genetic mouse model of cardiac CaMKII inhibition is protected from maladaptive remodeling caused by excessive βAR stimulation [76].

### 3.3. Arrhythmic Syndromes

The activation of CaMKII by acting on ion channels is described as a possible trigger for some inherited cardiac arrhythmia syndromes [75]. The overactivation of CaV1.2 by CaMKII results in an enhanced peak and a slowed Ca^2+^ inward current inactivation, causing membrane depolarization and the prolongation of the action potential duration, leading to arrhythmias [81]. CaMKII is also able to regulate both SR Ca^2+^ uptake and release. First, CaMKII catalyzes PLN phosphorylation [111], thereby reducing its inhibitory effect on Sarco-Endoplasmic Reticulum Ca^2+^ ATPase 2a (SERCA2a) thus causing the increase of Ca^2+^ reuptake by the SR and myocardial relaxation. Second, CaMKII phosphorylates RyR2, leading to pro-arrhythmic abnormal diastolic Ca^2+^ release from the SR, which results in Na^+^/Ca^2+^ exchanger forward-mode activity and afterdepolarization. Moreover, CaMKII inhibition drastically reduces diastolic SR Ca^2+^ leak in human and rodent cardiomyocytes [112], leading to decreased spontaneous Ca^2+^-release (arrhythmogenic event) and enhanced ability of the SR to accumulate Ca^2+^.

Notably, cardiokines, and in particular TNF-α and IL-1, can favor arrhythmias by increasing calcium currents, thus interfering with Ca^2+^ homeostasis and triggering arrhythmic events [113,114].

CaMKII activity has also been linked to atrial fibrillation (AF) in connection with the AMP-activated protein kinase pathway, leading to apoptosis and atrial remodeling [115,116]. Indeed, several inflammatory markers such as IL-6, C-reactive protein, and complementary factors are elevated in AF [117]. The acute administration of TNF-α in HL1 atrial cardiomyocytes leads to a significant increase in cytosol free Ca^2+^ levels [118]. Moreover, AF is associated with an increase of total, phosphorylated, and oxidized CaMKII [119,120], secondary to Ca^2+^ release from the SR [121]. 

TNF-α also promotes mitochondrial ROS production, which in turn leads to an enhanced oxidation of CaMKII [122]. Once active, CaMKII acts on downstream targets, such as ion channels (promoting arrhythmias) and pro-fibrotic pathways (promoting atrial remodeling), and mitochondria (promoting ROS-induced cell death). Moreover, animal studies showed that CaMKII inhibition is protective against AF [123]. The central role of CaMKII in the pathophysiology of AF makes it an attractive therapeutic target.

### 3.4. Influence of Cardiac Therapies on CaMKII Activation

CaMKII is a downstream target of multiple agonists for which effective antagonists are available and already used as routine clinical practice for cardiac diseases. These include ranolazine, ivabradine, beta-blockers, angiotensin-converting enzyme inhibitors (ACEI), and aldosterone antagonists.

Late Na^+^ current dysregulation in hypertrophic cardiomyopathy is responsible for the intracellular Ca^2+^ accumulation and activation of CaMKII [124]. Acute ranolazine administration reduces both the intracellular Na^+^ and Ca^2+^ levels and CaMKII activity, thus contributing to the reduction of hypertrophic cardiomyopathy-related cardiac remodeling myocardial dysfunction [125].

The pharmacological treatment of cardiac hypertrophy also includes the β-blockers, the renin inhibitors, and ACEI such as carvedilol, aliskiren and enalapril. Carvedilol exerts a beneficial effect based on its antioxidant, anti-inflammatory, and anti-fibrotic properties and significantly reduces CaMKII levels in isoproterenol-hypertrophied rats (also in combination with aliskiren treatment) [126,127]. Treatment with enalapril, in spontaneously hypertensive rats, is able to prevent hypertrophy, apoptosis, and CaMKII activity [128].

Furthermore, treatment with the mineralocorticoid receptor antagonist spironolactone reduces both ROS and ox-CaMKII levels in cultured neonatal myocytes stimulated with aldosterone, thus confirming CaMKII activity contribution to aldosterone-induced mortality during myocardial infarction [129].

It has been demonstrated that resveratrol has a cardioprotective effect based on its anti-inflammatory and antioxidant properties [130]. Resveratrol significantly prevents the diastolic intracellular Ca^2+^ increase, ROS production, and activation of CaMKII induced by H_2_O_2_ treatment in ventricular myocytes, which are overall responsible for stress-induced arrhythmogenic events [131]. The beneficial effect of resveratrol has also been demonstrated in a pressure overload model in which it exerts an anti-hypertrophic effect, increases cardiac systolic function, reduces interstitial and perivascular fibrosis, and prevents CaMKII activation [131].

## 4. Conclusions

Inflammation comprises a wide range of processes that affect many aspects of normal physiology and pathology. Inflammation switches from physiological to pathological mechanisms when it is not a time-limited event but a chronic process causing tissue damage or even death. The identification of possible modulators of the inflammatory response could be beneficial not only for chronic inflammatory diseases but also for the diseases in which the inflammatory component represents a limit to the resolution of the pathology, such as, in the cardiac scenario, myocardial infarction, pressure overload, I/R injury, and arrhythmic diseases. Increasing evidence suggests a pivotal role of CaMKII as a versatile kinase in many cardiac pathophysiological conditions involving inflammation. This is both a consequence of its activation properties in the presence of inflammatory states dysregulating Ca^2+^ balance, and to its ability to enhance the pro-inflammatory transcriptional signaling leading to inflammatory state amplification and persistence. Consequently, achieving a deeper knowledge of the mechanism by which CaMKII, the immune system, and inflammation are reciprocally modulated will be of potential therapeutic importance to mitigate the severity of many cardiac diseases.
